# Pushing the boundaries of innovation: the potential of *ex vivo* organ perfusion from an interdisciplinary point of view

**DOI:** 10.3389/fcvm.2023.1272945

**Published:** 2023-10-12

**Authors:** Jasper Iske, Andreas Schroeter, Samuel Knoedler, Timo Z. Nazari-Shafti, Leonard Wert, Maximilian J. Roesel, Felix Hennig, Adelheid Niehaus, Christian Kuehn, Fabio Ius, Volkmar Falk, Moritz Schmelzle, Arjang Ruhparwar, Axel Haverich, Christoph Knosalla, Stefan G. Tullius, Florian W. R. Vondran, Bettina Wiegmann

**Affiliations:** ^1^ Department of Cardiothoracic Surgery, Deutsches Herzzentrum der Charité, Berlin, Germany; ^2^ Charité-Universitätsmedizin Berlin, Corporate Member of Freie Universität Berlin and Humboldt-Universität zu Berlin, Berlin, Germany; ^3^ Department of General, Visceral and Transplant Surgery, Hannover Medical School, Hannover, Germany; ^4^ Division of Transplant Surgery, Department of Surgery, Brigham and Women’s Hospital and Harvard Medical School, Boston, MA, United States; ^5^ Division of Plastic Surgery, Department of Surgery, Brigham and Women’s Hospital and Harvard Medical School, Boston, MA, United States; ^6^ Department of Plastic Surgery and Hand Surgery, Klinikum Rechts der Isar, Technical University of Munich, Munich, Germany; ^7^ Department for Cardiothoracic, Transplantation and Vascular Surgery, Hannover Medical School, Hannover, Germany; ^8^ German Center for Lung Research (DZL), Hannover, Germany; ^9^ Lower Saxony Center for Biomedical Engineering, Implant Research and Development (NIFE), Hannover, Germany; ^10^ DZHK (German Centre for Cardiovascular Research), Partner Site, Berlin, Germany; ^11^ Department of Health Science and Technology, Translational Cardiovascular Technology, ETH Zurich, Zürich, Switzerland

**Keywords:** transplantation, transplantation heart, *ex vivo* organ perfusion, machine perfusion, *ex vivo* machine perfusion, organ modification, *ex vivo* surgery

## Abstract

*Ex vivo* machine perfusion (EVMP) is an emerging technique for preserving explanted solid organs with primary application in allogeneic organ transplantation. EVMP has been established as an alternative to the standard of care static-cold preservation, allowing for prolonged preservation and real-time monitoring of organ quality while reducing/preventing ischemia–reperfusion injury. Moreover, it has paved the way to involve expanded criteria donors, e.g., after circulatory death, thus expanding the donor organ pool. Ongoing improvements in EVMP protocols, especially expanding the duration of preservation, paved the way for its broader application, in particular for reconditioning and modification of diseased organs and tumor and infection therapies and regenerative approaches. Moreover, implementing EVMP for *in vivo*-like preclinical studies improving disease modeling raises significant interest, while providing an ideal interface for bioengineering and genetic manipulation. These approaches can be applied not only in an allogeneic and xenogeneic transplant setting but also in an autologous setting, where patients can be on temporary organ support while the diseased organs are treated *ex vivo*, followed by reimplantation of the cured organ. This review provides a comprehensive overview of the differences and similarities in abdominal (kidney and liver) and thoracic (lung and heart) EVMP, focusing on the organ-specific components and preservation techniques, specifically on the composition of perfusion solutions and their supplements and perfusion temperatures and flow conditions. Novel treatment opportunities beyond organ transplantation and limitations of abdominal and thoracic EVMP are delineated to identify complementary interdisciplinary approaches for the application and development of this technique.

## 1. Introduction

**Figure 1:**
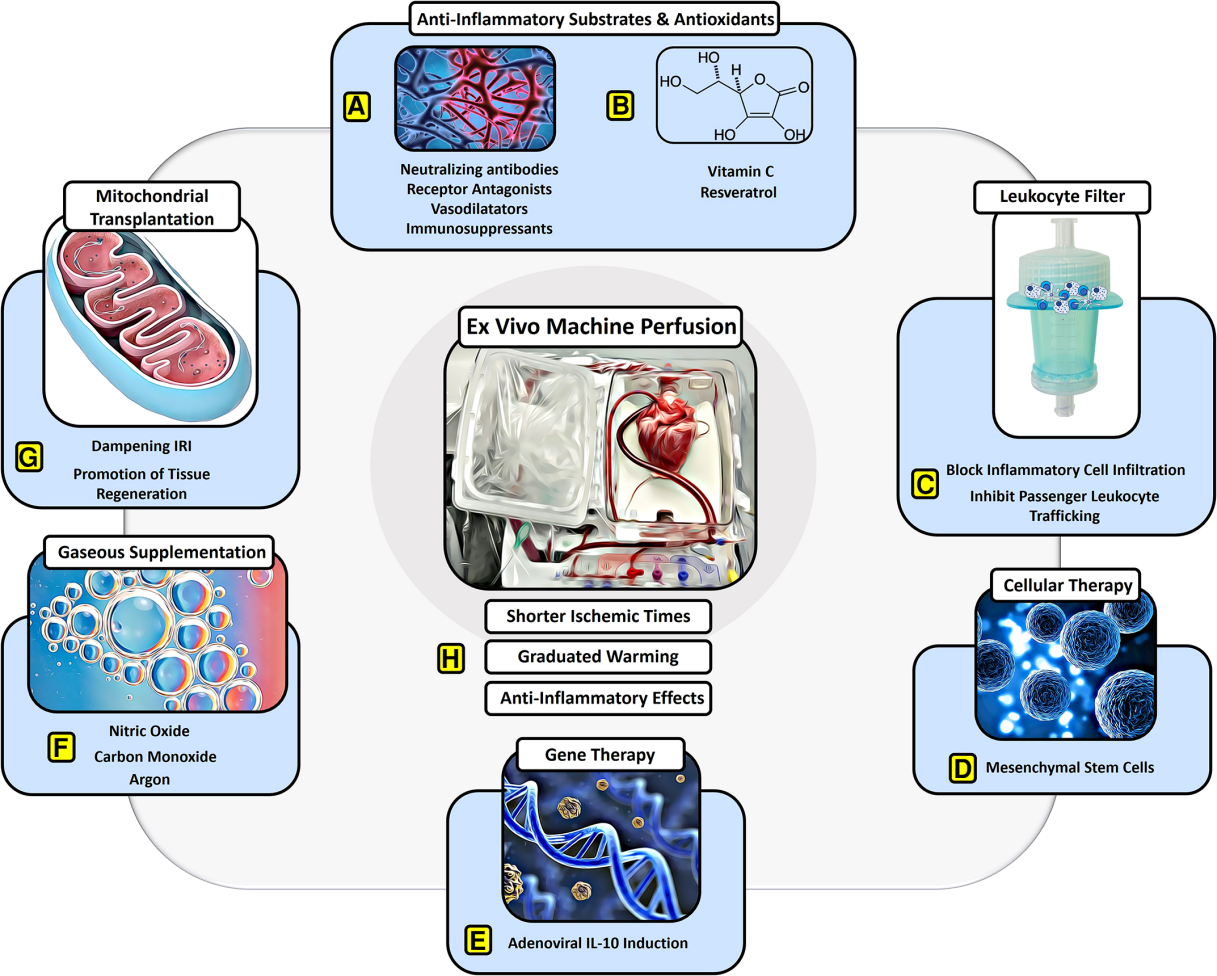
Protective effects of EVMP against ischemia–reperfusion injury. EVMP constitutes an interface to administer a broad range of therapeutic components that have focused on dampening IRI. Various (**A**) anti-inflammatory drugs and (**B**) antioxidants have been recruited to sufficiently dampen IRI-derived inflammation both experimentally as well as in human studies. (**C**) Moreover, a leukocyte filter can be recruited to deplete inflammatory cells and inhibit graft infiltration with ambiguous results. (**D**) Cellular therapies involving mesenchymal stroma cells have delivered promising results on dampening IRI during experimental EVMP. (**E**) Gene therapies targeting anti-inflammatory pathways such as IL-10 have led to a significant reduction of IRI during lung EVMP. (**F**) Gaseous supplementation during EVMP aims to promote vasodilation and compromise adverse physical effects on the microcirculation of perfused organs. (**G**) Mitochondrial transplantation constitutes a novel approach with beneficial effects on IRI in experimental settings for all organs used for transplant. (**H**) EVMP itself has been delineated to ameliorate IRI by reducing ischemic times, allowing for graduated warming of the organs and perfusion-derived anti-inflammatory effects.

The initial idea of isolated organ perfusion was first described in 1812. However, the potential clinical use was only investigated in detail after the introduction of solid organ transplantation to reduce ischemia–reperfusion injury (IRI) caused by the gold standard static-cold organ preservation (SCP). Nowadays, *ex vivo* machine perfusion (EVMP) enables organ perfusion with nutrition- and oxygen-enriched perfusion solutions in hypothermic to normothermic temperature conditions. These protocols led to significantly prolonged preservation times allowing for extended evaluation and potential reconditioning prior to transplantation. Moreover, EVMP dampened IRI associated with a reduction of alloimmune responses (1) (Figure 1). Several (pre-) clinical studies in abdominal and thoracic organ transplantation confirmed the significant benefits of EVMP over SCP, finally improving graft function and outcomes after transplantation (2–4). In addition, EVMP application resulted in significantly higher graft utilization rates due to an expansion of the procurement area by enabling long-distance organ transport (5) and the improvement of otherwise non-utilized marginal donor organs, particularly those retrieved from donors after circulatory death (DCD) and extended criteria donors (ECD) (6). These positive experiences paved the way for the continued implementation of EVMP as a daily routine and standard preservation technique for thoracic and abdominal organ transplantation.

In recent years, major efforts have been made to improve preservation strategies throughout all disciplines, employing EVMP not only for transplantation but also for a broadened field of applications. These developments focused on the extension of the organ-specific preservation time by optimized preservation protocols, including the improvement of perfusion solutions with the ideal composition of components (e.g., oxygen, nutrition, antibiotics), the perfusion temperature, and flow conditions (e.g., continuous vs. pulsatile) (7). Furthermore, standardized and validated tools for the assessment of organ function during EVMP were established and are currently the subject of investigation to enable the measurement of treatment success and predict post-perfusion outcomes (6). Based on these achievements, EVMP became available for novel treatment concepts, such as the reconditioning and modification of diseased organs *ex vivo*, and for specific tumor and infection therapies during surgical procedures. Furthermore, regenerative approaches in autologous settings are being explored, where patients are on temporary organ support while the diseased organs are being treated *ex vivo*, followed by reimplantation of the cured organ (8–10). In addition, EVMP has emerged as an important tool for preclinical research enabling *in vivo*-like preclinical pharmacological studies with the potential to accelerate the clinical transition of novel therapeutic approaches.

This review provides an interdisciplinary overview of the current abdominal and thoracic EVMP systems and their organ-specific preservation protocols followed by a summary of the relevant EVMP applications beyond organ preservation for allogeneic transplantation. The interdisciplinary application of this new technique may pave the way for researchers to go beyond the boundaries of their own professional discipline, learn from each other, and introduce new ideas in research and clinical practice for the benefit of the patients.

Finally, the authors are aware that a shift of paradigm is currently performed concerning the formerly used term *ex vivo*, which should nowadays only be used when talking about organs from living organisms, e.g., in the case of autologous reimplantation of *ex vivo* cured organs. In the context of allogeneic transplantation, *ex situ* should be used instead, as the organs are recruited from brain-dead or circulatory dead donors. Nevertheless, for better readability, the authors agreed to continuously use *ex vivo* throughout the manuscript.

## 2. *Ex situ* machine perfusion

### 2.1. General components and technique

Starting from the simple isolated and research-oriented organ perfusion in the 19th century, the continuous development of medical technology nowadays facilitates the routine application of *ex vivo* perfusion for solid allogeneic organ transplantation. Although a broad range of preservation protocols involving EVMP for different organs have been developed, all *ex vivo* perfusion systems consist of overlapping, general components. These components are combined harmoniously with each other but can be monitored separately to secure the base circulation providing a “near-physiological” condition.

The organ chamber contains the respective organs connected by vascular ports to the perfusion system. The organ is perfused by an integrated centrifugal or roller pump that ensures either a continuous or pulsatile blood flow via the arterial inflow cannula while blood is drained via the venous outflow tract. The downstream reservoir collects the circulating perfusion solution, also facilitating the optical control of a sufficient level of liquid to avoid air aspiration. Through the heater/cooler system, the perfusion solution is constantly tempered hypo-, subnormo-, or rather normotherm, depending on the underlying perfusion protocol. The supplement-enriched perfusate guarantees a nutrition supply, while the oxygenator provides the required oxygenation and decarboxylation of the perfusate as a prerequisite for sufficient cell metabolism. To compromise IRI and alloimmune responses and allow removal of accumulating toxic metabolites during *ex vivo* perfusion, the integration of leukocyte filters has been tested for several organs. However, its efficacy remains unclear as no differences in proinflammatory cytokines and leukocytes or clinical outcome parameters could be observed, probably due to rapid saturation of the filter with donor leukocytes as examined in porcine lung EVMP (11). Although all *ex vivo* perfusion systems operate as closed systems, ports at various locations are incorporated for additive supply and perfusate replacement or sample collection. Finally, a monitoring unit controls not only general (e.g., venoarterial pressures) but also organ-specific features (e.g., heart rate).

### 2.2. Organ-specific perfusion systems

To fulfill the “near-physiological” environment for the respective *ex vivo* perfused organ, distinct additional components are included in the basic circulation and are realized in various organ-specific *ex vivo* perfusion systems, which are explained below and summarized in Table 1.

**Table 1:** Established EVMP systems for experimental and clinical use for each organ.

Organ	System	Application	Perfusate solution	Perfusion temperature	Pros	Parameters for *ex vivo* monitoring
Kidney	LifePort Kidney Transporter (Organ Recovery Systems)	In clinical and experimental use	KPS-1®	HMP	Lightweight, portable, continuous monitoring, unaccompanied transport, ultrasonic detector to prevent air from entering the vasculature, SCS backup, pressure-controlled pump	Perfusion pressures, renal blood flow, temperature, vascular resistance
	Waters RM3 (Waters Medical Systems)	In clinical and experimental use	Any certified machine perfusion preservation solution eligible for pulsatile flow	HMP	Pulsatile perfusion, dual perfusion possible	Perfusion pressures, renal blood flow, temperature
	Kidney Assist (XVIVO Perfusion)	In clinical and experimental use	Any certified machine perfusion preservation solution	NMP, SNMP, HMP	Pulsatile perfusion, choice of setting the preferred perfusion temperature	Perfusion pressure, renal blood flow, temperature, reservoir temperature, vascular resistance
	WAVES System (Groupe-IGL)	In clinical and experimental use	WATERS IGL® Pulsatile Perfusion Solution	HMP	Lightweight, portable, pulsatile perfusion, unaccompanied transport	Perfusion pressure, renal blood flow, temperature, vascular resistance
Liver	Organ Care System™ Liver (TransMedics, Inc.)	In clinical and experimental use	OCS solution with RBC	NMP	Portable, approved for DBD and DCD livers	Hepatic artery flow (HAF), portal vein flow (PVF), oxygen saturation (SvO_2_), hematocrit (HCT), temperature, Hepatic Artery Pressure (HAP), and Portal Vein Pressure (PVP)
	Metra® (OrganOx)	In clinical and experimental use	Any certified perfusion solution compatible with OrganOx guidelines	NMP	Portable, allows perfusion for up to 24 h and a “back-to-base”-mode (NMP following initial SCP)	HAF, PVF, pH, lactate clearance, bile production
	Liver Assist (Organ Assist)	In clinical and experimental use	Any certified machine perfusion preservation solution	NMP HMP/HOPE/D-HOPE	Enables perfusion at every temperature between hypothermic and normothermic, pulsatile arterial and continuous venous flow, automatically adjusts the flow to the natural resistance of the graft	Perfusion time, flow, pressure, temperature, reservoir temperature, vascular resistance Cf-miRNAs from perfusate and bile samples have been used to assess graft viability and function
	Medtronic Portable Bypass System (PBS®)	In clinical and experimental use	Vasosol machine perfusion solution	HMP	Already established in cardiopulmonary bypass and extracorporeal membrane oxygenation	Perfusion time, flow, pressure, temperature, reservoir temperature, vascular resistance
Lung	Organ Care System™ Lung (TransMedics, Inc.)	In clinical and experimental use	OCS solution with RBC	NMP	Portable, potential use for split lung preservation, and *ex vivo* surgery, pulsatile flow, oxygenator can also be used to deoxygenize the perfusate and thus evaluate the oxygenation capacity of the graft	Flow (PF), pressure (PAP), VR, temperature, SaO_2_, SvO_2_, HCT, PAWP, PEEP, RR, TV
	XPS™ (XVIVO Perfusion)	In clinical and experimental use	STEEN Solution™	NMP	X-ray and CT-scan possibilities, in-line gases with real-time tracking (pO2, pH), separate sterile area and perfusionist non-sterile area	PA and LA pressure, temperature, flow, pH, pCO_2_, pump speed
	Lung Assist™ (Organ Assist)	In clinical and experimental use	Any certified machine perfusion preservation solution	NMP	Evaluation in hypothermic and normothermic conditions, compatible with any ventilators, enables perfusion at every temperature between hypothermic and normothermic, portable	Perfusion time, flow, pressure, temperature, reservoir temperature, vascular resistance
Heart	Organ Care System™ Heart (TransMedics, Inc.)	In clinical and experimental use	OCS solution with RBC	NMP	Portable, allows for DCD donations, accepting marginal hearts	Flow (pump, AOF), flow (CF), temperature (temp), pressure (AOP, PAP), heart rate, hematocrit (HCT), saturation (SvO2)
	Heart Box (XVIVO Perfusion)	In clinical and experimental use	XVIVO Perfusion Solution	HMP	Non-ischemic heart preservation (NIHP), used in experimental and clinical xenotransplants	Flow (CF), pressure (AOP, PAP)

#### 2.2.1. Kidney systems

In general, a urine reservoir, ports to extract perfusate or urine for assessing graft function, and special cannulas for potential abnormal kidney vasculature are organ-specific components for kidney machine perfusion devices. After the first clinical feasibility study demonstrated safe and promising outcomes in ECD using an EVMP system with a pediatric cardiopulmonary bypass technology (Medtronic) (12), several kidney EVMP systems have been developed, which are currently in clinical use. These involve the LifePort Kidney Transporter (Organ Recovery Systems), Waters RM3 (Waters Medical Systems), Kidney Assist (XVIVO Perfusion), and WAVES System (IGL Group). Although several perfusion solutions are under investigation, the only clinically proven fluid for kidney hypothermic machine perfusion (HMP) is Kidney Perfusion Solution-1 (KPS-1®).

The LifePort Kidney Transporter can be used for both pulsatile and non-pulsatile perfusion at 1°C–5°C and is portable with the possibility of unaccompanied transport, thus reducing logistical efforts and costs. It can be used with any certified machine perfusion solution eligible for HMP. Investigating the clinical benefits of the system, a reduced risk of delayed graft function, and an improved graft survival in the first posttransplant year when compared to SCS were shown (13). As of today, the LifePort Kidney Transporter is the most used perfusion device for clinical kidney HMP.

The Waters RM3, in turn, is a portable system that provides pulsatile flow for HMP at temperatures between 3°C and 8°C and can be used with any certified perfusion solution eligible for pulsatile flow. In addition to single kidney perfusion, simultaneous perfusion of two explanted kidneys is possible, and the system comes with trident adapters for the cannulation of grafts with anatomical anomalies, such as multiple renal arteries. Experimental dog studies comparing the flow-driven RM3 with the pressure-driven LifePort found no significant differences in transplant outcomes (14).

Another device for kidney EVMP, i.e., the Kidney Assist (XVIVO Perfusion), allows for pulsatile perfusion at a flexible temperature range (12°C–37°C), thus representing the only device capable of kidney normothermic perfusion (NMP). It is FDA-approved and can be used with any certified machine preservation solution, but it is non-portable. Clinical feasibility and safety have been shown with comparable outcomes for both oxygenated and non-oxygenated perfusion (15).

At least, the WAVES System (IGL Group) provides pulsatile HMP (2°C–8°C) using the WATERS IGL® Pulsatile Perfusion Solution. It is portable and designed for unaccompanied transport. Clinical safety has been reported with improved functional outcomes of machine-perfused kidney grafts (16, 17). Of note, it can also be used for combined kidney–pancreas preservation.

#### 2.2.2. Liver systems

Organ-specific modifications of liver perfusion systems include a bile reservoir and a second influx cannula for portal vein perfusion. Currently, three distinct EVMP devices for liver preservation are available for clinical use, namely, Liver Assist (Organ Assist), Organ Care System™ Liver (TransMedics, Inc.), and Medtronic Portable Bypass System (PBS®).

The Liver Assist (XVIVO Perfusions) is the most used EVMP device for liver perfusion that is compatible with any certified machine preservation solution. Providing pulsatile flow at temperatures between 12°C and 37°C, it represents the only currently available device capable of liver HMP or hypothermic oxygenated machine perfusion (HOPE). However, it is non-portable, thus requiring a combinatorial approach with other preservation strategies. Several clinical studies have shown improved transplant outcomes and reduced graft injury in HOPE-treated DCD (18–20) and ECD (21) organs and beneficial effects of NMP and combinatorial approaches (22, 23). The portable OrganOx Metra® allows for extended preservation times of up to 24 h during NMP. The biggest RCT on NMP demonstrated a 50% reduction in discard rates and a 50% lower level of graft injury when compared to CSP (24).

The Organ Care System™ Liver (TransMedics, Inc.) is also portable and uses OCS solution with red blood cells (RBCs) for pulsatile NMP at 34°C. The recent PROTECT trial comparing OCS preservation with SCS found reduced posttransplant allograft dysfunction and biliary complications and an increased use of DCD organs in the OCS group (25). It is FDA-approved for both DCD and DBD donor livers.

The PBS® (Medtronic), originally designed for cardiopulmonary bypass or extracorporeal membrane oxygenation, has also been used for clinical liver HMP (4°C–6°C). It provides pulsatile flow while utilizing Vasosol Organ Perfusion Solution. Clinical studies on the PBS demonstrated a reduction of proinflammatory cytokine production (26). However, there is a lack of recent experimental or clinical data.

#### 2.2.3. Lung systems

For lung EVMP, specific components include a respirator for lung ventilation during machine perfusion, enabling also for different ventilation modes, and an additional port allowing for bronchoscopy. This is based on experimental evidence that has shown the beneficial effects of continuous mechanical ventilation during machine perfusion. In porcine experimental animal models, mechanical airway pressure release ventilation following donation after circulatory death has been shown to reduce lung injury with improved oxygenation and compliance (27) while flow-controlled ventilation preserved alveolar recruitment (28). Moreover, preclinical data show that EVMP can also be used conversely in this setting to deoxygenate the perfusate, thereby assessing the oxygenation capacity of the lungs (29). Other experimental approaches such as airway pressure release ventilation and negative pressure ventilation have also been studied in experimental lung EVMP and were associated with improved pulmonary function (27) and reduced lung injury (30), respectively.

Currently, there are three systems for clinical lung EVMP available including the Organ Care System™ Lung (TransMedics, Inc.), XPS™ (XVIVO Perfusion), and Lung Assist™ (Organ Assist). Although all systems use NMP for graft preservation in clinical settings, they differ in some technical parameters and the utilized perfusate.

The Organ Care System™ Lung uses an OCS solution containing RBCs with an open left atrium (LA) and is the only portable device. It uses pulsatile perfusion for NMP at 34°C–37°C. Clinical safety and non-inferiority compared to SCS have been proven in the INSPIRE trial (5). Moreover, the OCS Lung™ has been evaluated for split lung preservation and *ex vivo* surgery (31). FDA approval has been granted for both standard criteria donor and ECD lung preservation, thereby including both DBD and DCD organs.

The XPS™ system (XVIVO) is non-portable and operates with STEEN™ solution and a closed LA while providing continuous NMP at 35°C–37°C. The NOVEL trial demonstrated the clinical safety and efficacy of the system (32). Of note, the XPS™ has been designed to allow radiographic imaging, thus facilitating x-rays and CT scans of the graft during perfusion (33).

The Lung Assist™ device is compatible with any certified machine preservation solution and operates with the LA being closed. It is also non-portable and provides pulsatile flow. Of additional interest, it allows for isolated in and *ex vivo* perfusion in both hypothermic and normothermic conditions (12°C–37°C). The ventilator is not included in the device, yet any pre-existing ventilator is compatible for use.

#### 2.2.4. Heart systems

In addition to other organs, heart EVMP also demands for organ-specific modifications of the machine perfusion system including cables for defibrillation or pacing. Two machine perfusion systems for the heart are currently in clinical use including the Organ Care System™ Heart (TransMedics, Inc.) and the Heart Box (XVIVO Perfusion).

The Organ Care System™ Heart is portable, uses NMP at 34°C–37°C, and provides pulsatile flow to the graft while being perfused with OCS solution containing RBC and donor blood. In 2015, a non-inferiority study showed non-inferiority compared to SCS preservation and paved the way for clinical application (34). Moreover, clinical studies revealed that the OCS enables heart transplantation from extended criteria DBD (35) and DCD (36, 37) donors. In 2021, FDA approval was granted for organs from DBD donors and, most recently, also for DCD organs, making it the currently only FDA-approved device.

The Heart Box (XVIVO Perfusion), in contrast, uses HMP at a temperature of 8°C and perfuses the heart with an oxygenated cardioplegic nutrition–hormone solution and ABO-compatible packed red cells. It is portable and provides continuous flow. The first-in-human study published demonstrated the feasibility and safety of this preservation technique in clinical heart transplantation (38) and a multicenter clinical trial that started in 2020 (NCT03991923). Of note, the Heart Box has most recently been used in the first xenogeneic pig-to-human xenotransplant (39).

### 2.3. Perfusion solution

Under (sub)normothermic conditions, perfusion solutions enable the preservation of organs in a pseudo-physiological environment with adequate oxygen, nutrients, and metabolic supply. Perfusates are required to balance cellular hydration and electrolyte homeostasis for edema prevention and also to reduce free radical peroxide scavengers to minimize oxidate injury (40). However, to date, the optimal perfusion characteristics and perfusate compositions for EVMP modes remain undefined and therefore lack standardization. This vagueness is mainly due to the variability of EVMP system application, duration, temperature, and flow conditions. Accordingly, a broad spectrum of solutions with different cellular compositions and additives is available.

#### 2.3.1. Base perfusion solutions

Perfusates are categorized into extracellular (i.e., high-sodium and low-potassium composition) and intracellular (low-sodium and high-potassium formula) solutions. Both variants have been successfully tested in EVMP studies, with their superiority seemingly depending on the case- and organ-specific conditions (41). Of additional importance, the composition of base perfusion solutions may fluctuate depending on the set temperature. Therefore, it is important to ensure correct temperature levels during the entire perfusion process (42). Although the safety and feasibility of extracellular-like Ringer's lactate have been demonstrated in human clinical trials of EVMP (43), the University of Wisconsin (UW) intracellular-type solution also has its raison d'être in organ preservation. When using these solutions clinically, perfusion temperatures were maintained at 34°C and 21°C, respectively (8, 44). More recently, the Institut Georges Lopez (IGL-1) solution emerged as an alternative to UW, featuring lower viscosity and potassium levels and replacing hydroxyethyl starch (HES) with polyethylene glycol (PEG) as an oncotic agent. This solution was used at a temperature of 4°C–6°C (45) (NCT01317342).

The XVIVO Göteborg STEEN Solution is a buffered extracellular solution with well-documented value in the field of lung and liver EVMP and is used at a temperature of 37°C (46). It includes human serum albumin and dextran to provide strong osmotic pressure and coat the endothelium from leucocyte interaction. As such, STEEN perfusion and circuitry have been found to maintain organ stability and functionality—even during prolonged EVMP (46). Notably, this solution can be supplemented with RBCs or remain acellular (41). The armamentarium of perfusates furthermore involves a wide array of modifications, such as the Custodiol-MP histidine–tryptophan–ketoglutarate (HTK) solution with high-flow, low-potassium, and anti-nitrosative/oxidant properties designed for oxygenated EVMP at 4°C (47), the cellular Organ Care System solution with a low-potassium dextran formula (at 37°C) (48), Perfadex as an extracellular and dextran-based electrolyte preservation solution (at 37°C–38°C) (49), or the Celsior solution as a colloid-free extracellular-type solution (at 2°C–8°C) (50). Next to the abovementioned preservation solutions, various others have been described (8, 51, 52).

#### 2.3.2. Cellular and gaseous composition

While hypothermic MP can be conducted with or without active oxygenation, in normothermic MP, adequate oxygenation remains vital and can be delivered either by RBCs, synthetic oxygen carriers, or diffused oxygen by carbogen gas mixtures (53). Since whole blood-based perfusates may exert pathogenic effects deriving from hemolysis and residual blood components including cells, complement, and inflammatory factors (54) while also being associated with logistic hurdles and limited supply (55), leukocyte-/thrombocyte-depleted and plasma-free perfusates have gained popularity in preclinical and clinical studies (12, 56–58).

In most studies, red blood cell-based perfusion solutions have been used. Such perfusates are known to efficiently transport oxygen while their constant flow can mitigate shear stress (59). However, blood-based solutions inherently harbor the risk of infection transmission and transfusion-related incidents including hemolysis. Therefore, a variety of alternatives have been proposed ranging from artificial oxygen carriers such as polymerized bovine hemoglobin-based oxygen carriers and pyridoxylated bovine hemoglobin to acellular oxygen-carrying media such as STEEN (60, 61). These modern solutions also offer the advantage of convenient storage and transport—at similar effectiveness and rheological-hemodynamic characteristics (62, 63).

Uniquely, cell-free perfusates allow for gradual rewarming of the graft from hypothermia to normothermic conditions. This advantage is significant since the increase in metabolic rate, which is associated with the abrupt restoration of normothermia, is postulated to be a secondary cause of IRI (64, 65). While mixtures with supraphysiological concentrations of oxygen are commonly implemented in EVMP protocols, hyperoxemia and varying oxygen tensions warrant further investigations, particularly when combined with acellular perfusates (41). Interestingly, hydrogen sulfide has been identified as a potential additive to induce a hypometabolic state and reduce oxygen consumption, thereby paving the way for the use of normoxic mixtures (66). Further gaseous supplementation may involve carbon monoxide, which was found to promote vasodilation and reduce IRI (67, 68), or argon, which is currently being investigated (69).

#### 2.3.3. Supplementary substrates

A potpourri of supplementary and modifiable components can be blended into the perfusion solutions, to mimic normal metabolism and recreate a near-physiological milieu. Additives that have been investigated include metabolic substrates, buffers, oncotic agents, anticoagulants, vasodilators, antioxidants, anti-inflammatory molecules, and hormones. Substrates for energy metabolism and nutrients, for example, are essential to perpetuate cellular metabolism during perfusion, thus enhancing cell viability. Additives such as glucose 5% or insulin are popular for all types of EVMP. In addition, pyruvate has been investigated as a metabolic substrate in cardiac EVMP and was found to enhance myocardial metabolism (70). Moreover, buffering agents are essential to maintain near-to physiological pH levels, since variations have been observed to adversely affect other physiological parameters such as pCO2 and HCO3^−^(71). Sodium bicarbonate and calcium gluconate, for example, may serve as universal pH and calcium buffers. Oncotic agents are included in various organ preservation solutions with the rationale of limiting tissue edema and subsequent cell death. Molecules such as HES and PEG have been used and could have further beneficial effects such as mitochondrial and glycocalyx protection (72). In addition, mannitol 10% is a well-established cross-organ applicable ingredient to elevate osmolality (41).

Blood-based perfusates are readily supplemented with anticoagulants to prevent clotting within the tubing lumen and decrease the thrombosis risk. For this purpose, the perfusate is usually heparinized or mesh-filtered (8, 41, 73, 74). Furthermore, nitric oxide (NO) levels are reduced during reperfusion, causing vasoconstriction and ultimately leading to prolonged cellular ischemia and aggravated necrosis (75). For this reason, vasodilators such as verapamil or prostacyclin can be applied to offset the transient vessel constriction upon reperfusion (76). Of note, the value of such medication [i.e., smooth blood (micro)circulation and organ perfusion] in acellular perfusates is yet to be defined (41, 77). Interestingly, in cell-free solutions, the biopolysaccharide dextran has emerged as an essential ingredient, preventing pathological leukocyte–endothelial interaction via antithrombotic properties and protecting the integrity of endothelium-rich organs. Thus, the addition of dextran to the perfusate may contribute to healthy vasculature and stable organ functionality (55). Antioxidants and anti-inflammatory molecules have also been under extensive investigation as supplements since they scavenge reactive oxygen species (ROS) arising from IRI and dampen the immunological response (78). As such, various agents including vitamin C, quercetin, and resveratrol have shown beneficial effects (79, 80). In addition, it is worth mentioning that vitamin C also improves microcirculation and reduces inflammation during EVMP. However, clinical benefits remain controversial (81). Hormones represent another group of potential additives with wide-ranging functional properties (82). In experimental liver NMP, for example, melatonin has been found to prevent oxidative stress and improve vascular conductivity (83). Moreover, dopamine reduced histological signs of damage and improved bile production (84). Other hormones investigated include erythropoietin and glucagon (82, 85, 86). Furthermore, EVMP provides a potential avenue for the administration of therapeutic drugs including chemotherapeutics or antibiotics, antivirals, and antimycotics, to decrease the microbial, bacterial, viral, and fungal load of infected organs and/or in the sense of prophylactic treatment (87). It is worth mentioning that the published protocols reveal a wide variance regarding the additives used. The supplements listed herein, therefore, represent only a selection.

### 2.4. Perfusion temperature

EVMP techniques can be classified according to the temperature applied during preservation roughly distinguished into cold, subnormothermic, and NMP that have found different implementations in the clinics depending on the organ of interest (Table 2).

**Table 2:** Different preservation strategies.

	HMP	SNMP	NMP
Temperature (°C)	4–10	20–32	37
Oxygenation	Both	Yes	Yes
Advantages	Elimination of debris, toxic metabolites, and free radicals Reduced endothelial damage, especially with pulsatile perfusion Collection of waste products	Damage from the cold is reduced Possible use as a resuscitation platform Drug administration is possible but inferior to NMP No need for oxygen carriers Collection of waste products	Metabolically active state No damage from the cold Superior for DCD and ECD grafts *Ex situ* graft assessment possible Novel interface for *ex vivo* drug therapies and bioengineering Longer preservation times are possible Collection of waste products
Disadvantages	Damage of the cold Reduced metabolism limits functional assessment Mitochondrial perturbations and endothelial activation Lower compliance in lung HMP Less suitable for DCD and ECD organs *Ex vivo* therapeutic interventions restricted	Metabolic activity is dampened Does not fully protect from reperfusion injury Less investigated	Higher costs and logistical effort High level of ATP depletion Risk of infection

#### 2.4.1. Hypothermic preservation

Hypothermic preservation at temperatures between 4°C and 10°C allows for the elimination of debris, toxic metabolites, and free radicals produced during hypothermia that would otherwise accumulate during cold static storage (88). First applied in kidney and liver preservation, the hemodynamic stimulation of the graft vasculature was found to compromise endothelial damage while pulsatile flow promoted vascular stress exerting beneficial effects on endothelial gene expression and function. Thus, HMP in both kidney (89) and liver (90) preservation has been found to enhance endothelial NO synthase (eNOS) phosphorylation, thereby preventing vasospasm while promoting NO-dependent vasodilatation at reperfusion.

However, due to the hypothermic state, the metabolic activity of the organ is dramatically impeded during perfusion restricting functional organ assessments (91). More importantly, hypothermically preserved grafts still sustain a cold ischemic injury through the inactivation of Na^+^/K^+^ pumps (92, 93). In kidney grafts, for instance, functional declines following HMP due to mitochondrial perturbations, decreased cell survival, and endothelial activation have been observed (94, 95). It is noteworthy that marginal donor organs from ECD and DCD donors have been delineated to be even more sensitive to cold ischemia (96, 97), particularly if the cold ischemic time (CIT) is prolonged (98). Therefore, the utilization of marginal kidneys remains limited while a significant prolongation of HMP preservation times is not feasible (99, 100). Supporting the evidence from kidney HMP, hypothermic preservation during lung EVMP was also associated with impaired metabolism and lower lung compliance (101). The application of *ex vivo* therapies as a promising approach during EVMP is thus restricted due to diminished exposure times of the graft during HMP and compromised pharmacodynamics at low temperatures. This may in contrast also promote the accumulation of the agent with harmful effects following reperfusion.

#### 2.4.2. Subnormothermic perfusion

Subnormothermic machine perfusion (SNMP) involves the preservation of explanted organs at 20°C–32°C and is currently undergoing experimental evaluation. In comparison with HMP, cold-induced graft injury is significantly reduced, while the augmentation of metabolic activity occurring during NMP is dampened at the same time. Thus, a metabolic state requiring additional oxygen carriers for adequate oxygenation is not reached (102). In experimental studies, the beneficial effects of SNMP on DCD grafts have been demonstrated (103). In kidney EVMP, for instance, subnormothermic perfusion at 22°C significantly reduced histological kidney damage and proinflammatory responses (103). Using SNMP in an experimental model of porcine liver EVMP improved endothelial cell function and bile duct injury (104), whereas oxygenated SNMP on human livers preserved liver function with minimal damage and sustained hepatobiliary parameters (44). In a rat DCD model of lung EVMP, subnormothermic perfusion at 28°C was associated with decreased proinflammatory cytokine expression and improved biochemical parameters such as compromised lactate and potassium levels and higher ATP and carbonylated protein levels (29). However, clinical data on the translational relevance of this procedure are sparse, and the Kidney Assist is the only commercially available device for SNMP.

#### 2.4.3. NMP

NMP allows organ tissue to remain metabolically active and precludes exposure to the cold, thus minimizing CITs. Graft preservation thereby enables normal cellular metabolism and recovery of ATP production in almost physiological conditions (58), whereas graft metabolites can be flushed, nutrient supply can be optimized, and microvascular circulation can be maintained. Therefore, NMP is considered the treatment of choice for marginal donor organs with successful clinical studies on the liver, lung, and heart (105–107), including DCD and ECD organs. Moreover, NMP of marginal kidneys has shown beneficial effects in porcine experimental models (108).

Furthermore, NMP is the only form of machine perfusion that enables pretransplant *ex vivo* assessment of the organ that could both alleviate decision-making in graft utilization and allow graft assessment during *ex vivo* therapy (109). Therefore, a broad range of viability criteria, such as lactate clearance, bile production, perfusate pH, glucose metabolism, flow rates, and perfusate transaminases, has been evaluated in liver EVMP (22, 110, 111). However, none of these parameters has been established as clinical guidelines because most studies are invariably based on small series with low case numbers despite being randomized or blinded. Larger collaborative studies that aim to confirm the potential biomarkers or shared databases allowing for the collation of obtained data are necessary and may support clinical translation.

Of further interest, previous studies on heart EVMP demonstrated the feasibility of utilizing solid-phase microextraction (SPME) microprobes with subsequent metabolomic profiling to uncover dynamic metabolic changes associated with organ injury and recovery (112), which may expand the range of parameters monitored during EVMP in future studies.

In addition to functional assessment, NMP constitutes a novel interface for *ex situ* therapies as pharmacokinetics and pharmacodynamics of drugs should not be altered by low temperature during HMP. Most relevantly, NMP has enabled a significant prolongation of preservation times throughout all organ types with 24 h in kidney (113), 1 week in liver (114), 3 days in lung (115), 24 h in heart (116) EVMP. Prolonged preservation times, in turn, provide the opportunity to perform *ex vivo* therapies that demand long application times including gene therapies and bioengineering and *ex vivo* surgery.

Of note, approaches combining NMP with HMP have also been tested in livers and demonstrated improved functional results (117).

### 2.5. Flow conditions

Flow conditions are crucial parameters of EVMP as they regulate graft supply with oxygen and nutrients and clearance of CO_2_ and metabolic products. In addition, flow conditions have been shown to influence the organ protective effects of perfusion solutions and mediate the occurrence of graft edema (8).

Considering the form of flow, pulsatile and continuous flow applications can be distinguished. Pulsatile flow during cardiopulmonary bypass, for instance, has been found to significantly improve vital organ recovery throughout several types of animal models associated with a preserved microcirculation when compared to continuous flow (118, 119). The pulsatile flow, in turn, generates vascular shear stress, which has been considered to influence endothelial gene expression and function (5, 38). Indeed, pulsatile pressure can enhance renal flow in isolated kidney perfusion, improving vascular conductivity that translates into increased clearance of creatinine, sodium reabsorption, and reduced tubular cell injury (120). Mechanistically, better vascular conductance upon pulsatile perfusion in kidneys could be attributed to improved endothelial release of NO and reduced secretion of endothelin-1 (121). However, studies comparing continuous to pulsatile perfusion in kidney pairs found no significant differences in graft survival and kidney function (7). In addition, an experimental study on porcine lungs reported no significant improvement in lung function parameters upon integration of a modified roller pump generating pulsatile flow (122). Taken together, clinically applicable evidence is scarce, and more research on flow forms is needed, especially considering temperature, perfusate, and the respective organ perfused. Although clinical studies in cardiopulmonary bypass patients indicate beneficial effects of pulsatile perfusion (118), it remains to be elucidated whether this also applies to clinical EVMP.

In addition to the form of flow application, the flow rate constitutes another important parameter for organ protective perfusion that has been mainly studied in lung EVMP. Several protocols using different percentages of the donor cardiac output or fixed flow rates exist including the Lund protocol (100% of cardiac output) (123), the Toronto protocol (40% of cardiac output) (124), and the OCS protocol (2–2.5 L/min) for lung EVMP (5). Since all studies investigating these protocols compare outcomes to SCS, no direct comparison between protocols can be made. Moreover, differences in study design, lung transplant type, and patient characteristics do not allow for statistically significant comparisons between these protocols (125). Noteworthy, experimental studies have also investigated lower flow rates comparing EVMP flows of 40%–20% in porcine DCD lungs. Intriguingly, improved lung function, reduced edema, and attenuated inflammation after transplant were observed when using flow targets of 20% (126). Supporting clinical evidence derives from studies comparing high-flow cellular to low-flow acellular machine perfusion, demonstrating higher transplant suitability, higher wet-to-dry ratio change, and decreased histological lung injury in the low-flow group (127).

## 3. Potential of EVMP beyond ordinary graft preservation

With a broad range of EVMP systems being available for both clinical as well as experimental applications, novel treatment concepts for both the allo- and xenogeneic environment are being explored. Of translational interest, significant efforts are also being made to investigate innovative approaches for the autologous setting, where the diseased organ will be treated *ex vivo*, while the patient is subjected to temporary organ support followed by replantation of the cured organ. In the meantime, the patient is subjected to temporary organ support such as hemodialysis for the kidney or the molecular adsorbent recirculating system for liver compensation and extracorporeal membrane oxygenation for mechanical heart–lung support (Table 1). Most notably, this procedure precludes complications deriving from disproportional organ size and the detrimental side effects of immunosuppression associated with allo- and xenogeneic transplantation while being timely limited only by the *ex vivo* preservation times.

### 3.1. Ultima-ratio drug therapies

As EVMP enables isolated *ex vivo* perfusion of the explanted organs, it provides a novel interface to treat them by high-dosage medication without the otherwise significant disadvantage of dose-limiting systemic side effects, resulting in more effective therapeutic success. In this context, EVMP has been established as a therapeutic platform to administer ultima-ratio therapies of failing organs in patients with otherwise poor prognoses and non-tolerable contraindications for systemic administration.

**Figure 2:**
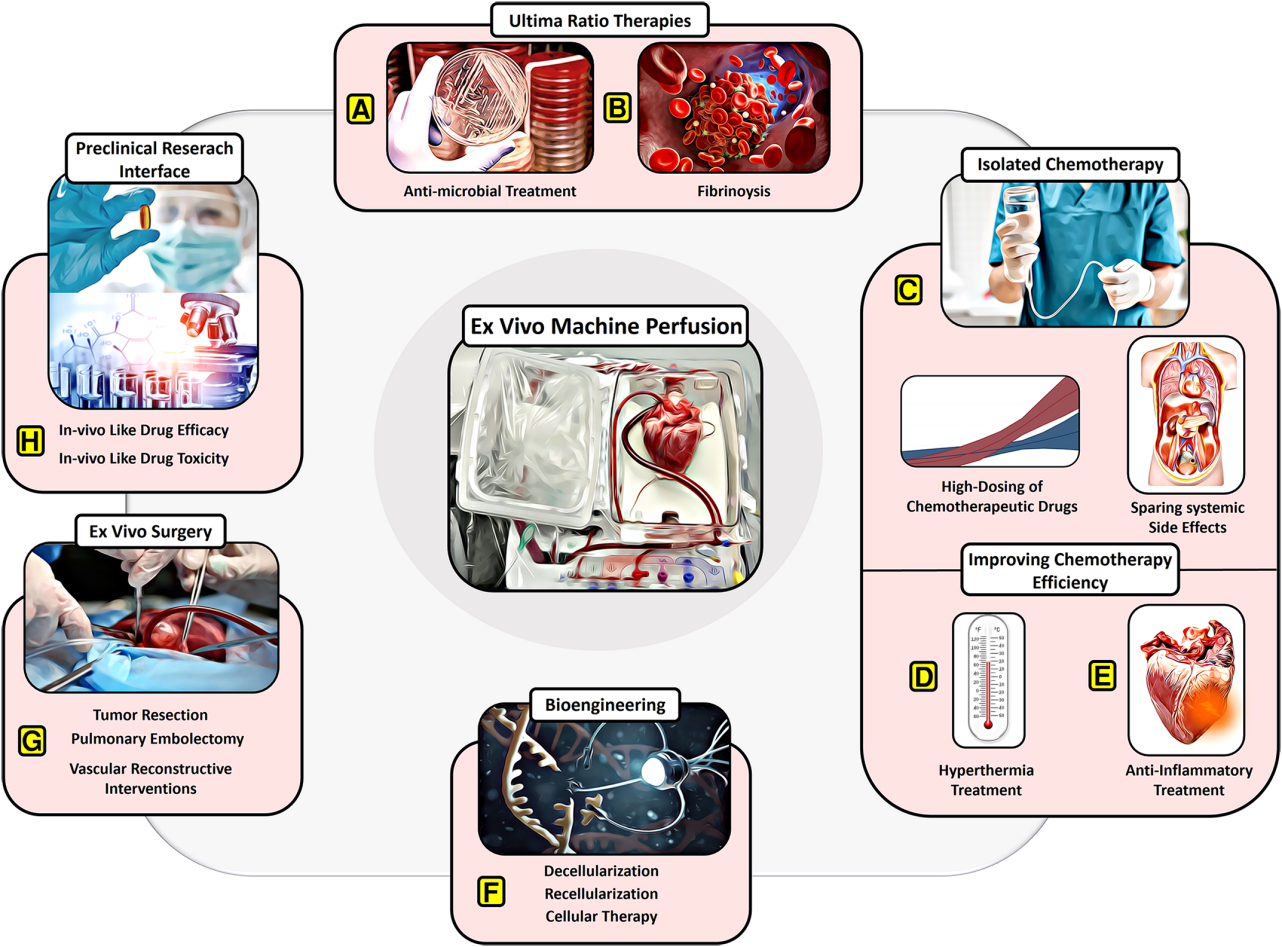
Potential of EVMP beyond transplantation. (**A**) Ultima-ratio therapies involving antimicrobial treatment for severe infections and (**B**) thrombolytic drugs for severe embolism. (**C**) Isolated chemotherapy allowing for high dosing of chemotherapeutic drugs despite systemic side effects. (**D**) Improving chemotherapy efficiency through recruitment of hyperthermic preservation or (**E**) anti-inflammatory therapies or hypothermic preservation. (**F**) Bioengineering approaches involving decellularization and recellularization. (**G**) *Ex vivo* surgery facilitating tumor resection, pulmonary embolectomy, and vascular reconstructive interventions. (**H**) Providing a preclinical research interface for *in vivo*-like drug testing.

This is of particular relevance for the lungs, as severe bacterial lung infections are one of the most frequent reasons for hospital mortality due to sepsis and systemic organ failure (128). Numerous competing factors in critically ill patients have been characterized to impede effective antimicrobial drug dosing required to eliminate pathogens including increased or decreased renal blood flow, renal and hepatic dysfunction, changing volume of distribution, and initiation of mechanical support devices such as continuous renal replacement therapy or extracorporeal membrane oxygenation (129). Thus, high-dosage antimicrobial agents during EVMP with subsequent re-transplantation could provide an option to achieve augmented in-organ doses of antimicrobial agents while sparing systemic side effects. Strikingly, subjecting explanted lungs infected with incurable, multidrug-resistant pseudomonas aeruginosa to a high dosage of colistin during EVMP enhanced overall survival in a porcine lung autotransplant model (130). Of note, colistin has been shown to exert tremendous side effects causing renal and neurological toxicity with higher cumulative doses, therefore limiting its effective *in vivo* application (131). Consistent with this study, a high dosage of empiric antimicrobial agents added to the EVMP perfusate of marginal donor lungs caused an effective reduction in microbial burden (132), improving pulmonary lung function with increased oxygenation, better pulmonary compliance, and reduced PVR (133). Moreover, isolating the infected organ from the organism for EVMP treatment concomitantly removes the source of infection restricting septicemia and associated systemic immune responses, which otherwise have been associated with accelerated multi-organ dysfunction, compromised antimicrobial drug efficiency, and death (134, 135). In order to further attenuate infectious organ injury during EVMP, novel cellular therapies involving mesenchymal stem cells (MSCs) are currently evaluated in preclinical and clinical trials. Thus, tracheal instillation of MSCs during EVMP of *E. coli*-injured human lungs increased bacterial clearance and dampened inflammatory infiltration and proinflammatory cytokine production while improving alveolar fluid clearance (136) (Figure 2A).

Allowing for isolated, high-dosage therapy without systemic distribution of thrombolytic drugs, EVMP may furthermore display an alternative treatment approach to surgical pulmonary embolectomy in patients with large-scale pulmonary embolism and significant contraindications for fibrinolysis. In support, a recent case report demonstrated the feasibility of thrombolysis during EVMP in a donor lung affected by dispersed embolization with improved paO_2_, dynamic compliance, and less pulmonary edema allowing for subsequent, successful transplantation (137) (Figure 2B).

High-dose application of therapeutic agents such as antibiotic drugs during EVMP demands biomonitoring tools to assess the tissue concentration of the drug. Therefore, novel methods such as *ex vivo* SPME coupled to liquid chromatography/mass spectrometry allowed for rapid quantification of doxorubicin in porcine lung tissue by inserting a microfiber for 20 min (138).

Considering that many agents with potential for EVMP application display short lifespans, further investigation to define the ideal time point and duration of therapy administration is required. Notably, a recent report demonstrated the utilization of anti-CD31 to enhance the delivery of nanoparticles to explanted human kidney endothelium during EVMP, which can serve as depots for long-term drug release ensuring organ-specific therapy continuation following reimplantation (139). Finally, EVMP could augment the potential of novel gene therapies. Although approved by the FDA within the recent decade, they are associated with a significant economic burden, often requiring multiple applications due to insufficient delivery (140, 141). Here, EVMP could uphold drug levels by isolated target organ perfusion without systemic drug distribution, which would significantly reduce costs and could lead to a more optimized therapeutic effect. Concomitantly, drugs that have shown promising effects, but whose clinical implementation had been hindered due to systemic side effects (142), could find new upwind through EVMP sparing systemic exposure.

### 3.2. Improved effectiveness of chemotherapy

At this point, EVMP usage focuses exclusively on the autologous setting, where organs will be treated *ex vivo*, while the patients are on temporary organ support, followed by the autologous replantation of the cured organ. In addition to the aforementioned high-dosage application of various medications, chemotherapeutics, hyperthermic conditions, and attenuated local organ inflammation may enhance the effectiveness of the chemotherapy.

#### 3.2.1. Improving chemotherapy efficiency by increased drug doses

Chemotherapeutic drugs display a dose-dependent efficacy on tumor cells in a broad range of cancer types. Thus, platinum-based chemotherapeutics, for instance, that are frequently administered in small-cell lung cancer, have been shown to achieve a significantly higher complete response rate, overall survival, and the number of 2-year survivors when applied at high dosages including cisplatin and carboplatin in contrast to carboplatin alone (143). However, side effects arising from high doses of chemotherapeutic regimens limit their efficacy, thus compromising outcomes of this therapy (144–146). In particular, platin doses are limited by kidney damage arising from acute tubular necrosis and proximal tubule apoptosis (147) and liver toxicity due to hepatocyte necrosis and perisinusoidal fibrosis (148). These systemic side effects could be spared during EVMP, while the lungs could benefit from high-dosage application with significantly improved therapeutic efficiency, thus providing an option for end-stage cancer patients. As a proof of concept, high dosing irinotecan with 20 times higher concentration (2,000 mg/L) than the concentration for systemic application, which engenders strong neutropenia and gastrointestinal toxicity in patients (149), did not induce drug-dependent reperfusion edema or toxic injury to the lung parenchyma (150) (Figure 2C).

Moreover, the tumor microenvironment has been shown to exhibit an unbalanced starling mechanism in addition to increased vascular permeability as well as a malfunctional lymphatic system inside the tumor mass, leading to an increased tumor interstitial fluid pressure (TIFP), which in turn impedes drug distribution in many tumors. A significant increase in the composition of the extracellular matrix furthermore compromises drug delivery. Administering additional drugs to the perfusion solution of EVMP to lower the TIFP or augment convection may allow an additional improvement of chemotherapeutic efficacy. Indeed, the angiotensin inhibitor losartan has been shown to reduce stromal collagen and hyaluronan production, therefore reducing TIFP and resulting in increased vascular perfusion potentiating chemotherapy (151). Moreover, blocking the VEGF receptor type 3 was found to decrease lymphatic drainage, thus compromising TIFP and consequent drug removal (152, 153).

#### 3.2.2. Improving chemotherapy efficiency by hyperthermic conditions

A complete explantation of the target organ with subsequent implantation into an EVMP system appears detrimental; however, unique features of modern EVMP systems may improve adjuvant therapies due to the opportunity to influence preservation parameters and the application of therapeutic agents to the perfusate solution. In particular, EVMP enables fine-tuned regulation of the perfusate temperature that could exert beneficial effects during cancer therapy. Indeed, hyperthermia has shown significant antitumor effects by affecting tumor growth directly and improving chemotherapy efficacy (42). Thus, hyperthermic treatment of cancer cells has been shown to induce the DNA damage response by promoting DNA strand breaks, histone g-H2AX foci formation, and ATM phosphorylation, while decelerating DNA replication and repair through downregulated DNA polymerase and topoisomerase activity (154). In the clinics, hyperthermia is already frequently combined with chemotherapeutic regimens leading to higher fluidity of the phospholipid bilayer in tumor cells, thus augmenting drug permeability. Consequently, cisplatin has been shown to exhibit synergistic effects with hyperthermia at 43°C on cell growth inhibition (155). During EVMP, hyperthermia can be easily induced and applied locally targeted to the preserved organ facilitating synergistic cancer treatment with chemotherapeutic drugs and hyperthermia. Further supporting this concept, local hyperthermia in addition to neoadjuvant chemotherapy augmented the effect of the etoposide, ifosfamide, and doxorubicin regimen on soft tissue sarcoma with higher treatment response rate, compromised local progression, and overall improved survival (156). In addition, regional inductive hyperthermia in patients with liver metastasis deriving from breast cancer increased the overall treatment efficacy with a 33.9% higher regression rate (157) (Figure 2D).

#### 3.2.3. Improving chemotherapeutic efficiency by dampening local organ inflammation

A broad range of chemotherapeutics including platin, taxanes, 5-FU, and doxorubicin have been delineated to promote a prominent proinflammatory tissue response with the expression of IL-6, IL-8, TNF-α, and INF-β, which in back turn impedes their efficiency and enables metastasis formation (158). Paclitaxel, for instance, induces augmented cytokine production including IL-6, which was mediated via TLR-4 in breast cancer cells. TLR4- expression, in turn, was correlated with conferring resistance to the drug by promoting anti-apoptotic proteins (159), while IL-6 was found to endorse tumor progression inducing angiogenesis and proliferation via the STAT-3 pathway (160). Inhibiting chemotherapy-derived inflammatory signaling has been shown to compromise drug resistance. Neutralizing IL-6 with antibodies, for instance, sensitized multiple tumor types toward distinct chemotherapeutic regimens (161, 162). Since EVMP provides an interface to administer anti-inflammatory agents, utilizing EVMP during *ex vivo* chemotherapy could restrain chemotherapy-derived organ inflammation, thus augmenting therapy efficiency. In support of this approach, the administration of various anti-inflammatory reagents has been tested in clinical EVMP studies showing significant amelioration of donor organ inflammation with compromised inflammatory cytokine expression including IL-6 which ultimately translated into improved function. In lung transplantation, for instance, inhibiting adenosine signaling with A2AR agonists during EVMP inhibited TNF-α, IL-1, and IL-6 expression (163). Similarly, IL-6 receptor blockade with tocilizumab (164), as well as melatonin administration (165) during EVMP, inhibited IL-6-derived IRI in cardiac transplantation. Moreover, in liver transplantation, administration of an anti-inflammatory cocktail comprising alprostadil, n-acetylcysteine, and carbon monoxide in addition to subnormothermic temperature during EVMP restrained TNF-α and IL-6 expression following transplantation (68). At least, the integration of a cytokine filter during lung (166) and kidney perfusion (167) reduced overall proinflammatory cytokine expression with diminished edema formation and improved blood flow, respectively.

In addition to compromising chemotherapy efficiency, the inflammatory response induced by chemotherapeutic drugs furthermore aggravates the function of the target organ. Cisplatin is commonly recruited to treat biliary cancer both in a neoadjuvant as well as palliative setting, thus co-exposing the liver to its toxicity. Notably, platin-based chemotherapeutics have been delineated to promote hepatic injury via oxidative stress and augmented inflammation leading to cellular necrosis (168–170) and organ fibrosis. In contrast, EVMP has been demonstrated to reduce oxidative stress and inflammation in preserved organs, which may in turn dampen chemotherapy-derived organ injury. Metabolomic profiling during liver EVMP, for example, revealed increased ATP levels as well as higher NADPH/NADP ratios associated with reduced lactate levels in liver tissue in the kinetics of 3 h of liver perfusion (171), which may also diminish platin therapy-related oxidative stress. Furthermore, administration of enkephalin, an *δ*-opioid agonist with antioxidative properties, during liver perfusion compromised oxidative stress and prevented mitochondrial dysfunction, resulting in higher ATP and glutathione in addition to lower AST and malondialdehyde levels in a rat liver EVMP model (172) (Figure 2E).

### 3.3. Bioengineering and organ modification

Bioengineering is considered an innovative and promising future approach with the potential to recondition diseased organs (173). Hereby, EVMP is proposed as a promising interface to deliver cellular products exclusively to the target organ. Strikingly, a recent study reported for the first time the successful engraftment of cholangiocyte organoids into the intrahepatic biliary tree during EVMP, while providing proof of concept that these organoids can repair injured bile ducts. In detail, red fluorescent protein (RFP)-expressing cholangiocyte organoids were injected into the terminal branch of the intrahepatic duct of human, ischemic injured livers at the start of EVMP. Subsequently, organoids exhibited expression of key biliary markers (KRT7, KRT1×9, CFTR, GGT) and improved bile production with increased pH and higher volume despite showing no trans-differentiation into other hepatic lineages (174).

In addition to treating diseased organs during EVMP with subsequent reimplantation, bioengineering is furthermore envisioned to enable the recreation of tissue parts for subsequent implantation as an alternative strategy to organ transplantation. Therefore, EVMP has been proposed as an interface for the decellularization and the recellularization of bioartificial organs under physiological conditions with subsequent implantation (175, 176). In general, decellularization was achieved in a broad range of organs during EVMP with preserved organ architecture and ECM components in addition to low levels of DNA and physiological abundance of glycosaminoglycans and chemical and mechanical components of the ECMs (177). Moreover, administration of human placenta-derived endothelial progenitor cells (EPCs) during EVMP was shown to induce successful recellularization with proliferative EPCs repopulating kidneys, lungs, and hind limb vascular intimae. Of note, a vascular chimerism with human EPCs lining the luminal surface of rat blood vessels, alongside rat cells within the tunica media and beyond, artificially generating vascular chimerism (176). In addition, various studies have demonstrated the effective decellularization of organs from different animals preserving extracellular matrix composition and architecture to engineer bioartificial organs via repopulation with human cell lines (173). Notably, infusing human endothelial cells *ex vivo* through the renal artery and vein of decellularized rat kidneys resulted in homogenous distribution in the vasculature compartments with site-specific endothelial specialization (178). Similarly, administering human perivascular and endothelial cells through the pulmonary artery and pulmonary vein in isolated and decellularized rat lungs and human lung lobes resulted in successful endothelial cell coverage with functional vascular lumen structures being detected (179). Underscoring the clinical feasibility of bioengineering, this study also examined the transplantation of the recellularized lungs into rats showing the formation of continuous, polarized vascular lumens that remained perfusable 3 days after transplantation (179). Similarly, transplanting clinically scaled porcine liver scaffold with human umbilical endothelial cells (HUVECs) revealed HUVECs localization within sinusoidal regions in addition to expression of a liver sinusoidal endothelial cell-like phenotype. Strikingly, subsequent heterotopic transplantation into immunosuppressed porcine recipients resulted in 15 days of continuous *in vivo* perfusion of the revascularized bioengineered liver (rBEL) (180) (Figure 2F).

Mitochondrial transplantation constitutes an additional and very novel approach to modify tissue homeostasis and diseased organs, which may be of translational relevance for EVMP-based regenerative therapies (181). Indeed, mitochondrial transfer has been demonstrated to improve IRI in a broad range of organs while also ameliorating pathological tissue dysfunction. Injecting mitochondria into the hearts of diabetic rats following IRI, for instance, resulted in the recovery of left ventricular function and a reduction of infarct size (182). Likewise, mitochondrial transplantation into the spleen improved liver function while administration via pulmonary artery vascular delivery or tracheal aerosol delivery improved lung mechanics and reduced lung tissue damage following IRI (183). Similar effects had been observed when performing mitochondrial transplantation through renal arteries with protective effects against renal IRI (184). Noteworthy, the effects of mitochondrial transplantation on organ reconditioning have also been delineated with isolated mitochondria of HepG2 cells injected into high-fat diet-fed mice, effectively improving non-alcoholic fatty liver disease (185).

Of relevance, most studies have indicated that the therapeutic effects of a single administration of mitochondria may be transient. Therefore, EVMP may allow higher doses and prolonged exposure to mitochondrial-carrying vectors while providing an interface to determine the time point and route of administration as well as the impact of repetitive cycles (186).

Bioengineering approaches using EVMP are also recruited to create immunotolerance to effectively counteract both the lifelong intake of immunosuppressive drugs and the risk of chronic rejection. One approach focused on the genetic modification of the MHC complex and the minor histocompatibility antigens (mHag) of the vascular endothelium. Notably, administration of short hairpin56 RNAs targeting beta-2 microglobulin and class II transactivator transcripts using lentiviral vectors during EVMP resulted in durable MHC I and II complex suppression without affecting cell viability or tissue integrity (187–189). Another approach utilized adenoviral vectors to induce IL-10 expression in donor lungs to prevent the development of primary graft dysfunction in a large animal survival model. Indeed, this approach was shown to be safe, to improve lung function, and to have an immunological advantage in both innate and adaptive immune responses (190).

### 3.4. *Ex vivo* surgery

Allowing for increasingly prolonged *ex vivo* preservation of organs, EVMP has paved the way for surgical *ex vivo* procedures. Utilizing stained perfusion solutions (e.g., methylene blue) in addition to altering flow conditions hereby allows for visualization and therefore suturing of even smaller leakages avoiding significant blood loss over time. Moreover, enabling the visualization and access to all organ sites and a prolonged period of time for accurate tissue preparation and reconstruction, this technique has raised significant interest throughout all disciplines utilizing EVMP.

For surgical management of renovascular diseases, *ex vivo* surgery is associated with significant technical advantages allowing for vascular reconstructive interventions. Thus, laparoscopic nephrectomy with subsequent autotransplantation was successfully utilized for renal artery aneurysms affecting distal vascular branches (191) and for nephron-sparing resection in the case of a large renal tumor (192). Integration of an EVMP system for *ex vivo* vascular kidney surgery, in turn, was associated with improved assessment of the perfusion characteristics of the remodeled kidney, in a study performing *ex vivo* surgery in patients with a solitary kidney and either dysplastic aneurysm, Takayasu disease, or fibrodysplasia lesions (193), thus preventing nephrectomy and lifelong dialysis.

Liver and intrahepatic bile duct cancers and hepatic metastases deriving from other extrahepatic tumors account for the most prevalent tumors in humans (194). Surgical resection hereby displays the gold standard therapy for most of these pathologies. However, compromised liver function and lesions at difficult anatomic sites, for instance, with the involvement of larger vessels constitute a contraindication for curative surgery. Since then, various studies utilizing *ex vivo* surgery to resect complicated hepatic malignancies including hepatocellular carcinoma, cholangiocellular carcinoma, and focal nodular hyperplasia and hepatic metastasis, achieving significant R0 resection rates (93,4%, CI: 81.0%–97.9%). However, these interventions had been associated with high 30-day mortality (9.5% CI: 5.9%–14.9%) (195). Integrating EVMP during *ex vivo* surgery could enable a prolonged preparation and aftercare during the operation in addition to ameliorated IRI upon reimplantation. Moreover, recruiting hyperthermia and *ex vivo* chemotherapy during surgery may further adjuvate tumor elimination. Of note, *ex vivo* liver resection has also been introduced as a curative approach for non-resectable, end-stage hepatic alveolar echinococcosis (HAE) associated with dissemination into the intrahepatic conduits and adjacent structures. Thus, a recent study reported a curative treatment in 29 of 31 patients with long-term recovery and no HAE recurrence (196).

The feasibility of EVMP-supported operations in thoracic surgery has been demonstrated in porcine models of large tracheobronchial leakage with successful implantation of a pericardial patch and replacement of the distal trachea with an aortic graft using the OCS (197). Moreover, EVMP has already been applied in the clinical setting with successful lung autotransplantation for centrally located and locally advanced lung cancer to spare lung parenchyma by avoiding pneumonectomy, which underscores the potential of this procedure (198). Noteworthy, integrating an EVMP system could furthermore enable the application of topical, high-dosage therapeutic drugs avoiding systemic side effects, which is of particular interest in supporting the long-lasting success of *ex vivo* tumor resections in lungs (199). Moreover, surgical procedures can be performed in the absence of ventilation improving surgical accuracy, which otherwise is hardly possible during in-human surgery, in particular in patients with threatening decreased lung function.

Exploiting the improved access routes to critically located tumors, various case studies in cardiac surgery have reported successful *ex vivo* resections of cardiac sarcoma (200), complex atrial myofibroblastic tumors (201), and giant large atria following chronic mitral valve disease on an *ex vivo* beating heart (202). Moreover, single centers also have reported on larger case numbers of cardiac autotransplantation with *ex vivo* tumor resection for malignant complex primary left heart tumors (203) (Figure 2G).

### 3.5. Preclinical in-human research

Being able to preserve solid organs in a perfused, physiological environment enables a new field for *in vivo*-like preclinical studies with the potential to accelerate the clinical transition of novel therapeutic approaches. Therefore, a large choice of animal models for kidney, liver, and thoracic organ EVMP have been developed, which simultaneously facilitate the investigation of therapeutic regimens for diverse disease models. However, multiple differences, for example, between rodent and human perfusion models restrict the translational relevance of these studies due to lower EVMP perfusion flow in rat models (204) and hypersensitivity toward dextran-based perfusion solutions, which does not occur in humans (205). Moreover, a broad range of diseases, in particular various malign tumor animal models, are still lacking substantial transferability with regard to the strong heterogeneity of tumorigenesis (206).

Utilizing organs from deceased patients in contrast enables the opportunity to test novel pharmacotherapeutic therapies in relevant human disease models and allows for more precise prediction of therapy efficacy when compared to animal models.

For lungs, acute respiratory distress syndrome (ARDS), for instance, represents an acute life-threatening pathology frequently deriving from severe infection, which evolves rapidly and confers high mortality on the afflicted (207). Advances in clinical care have significantly improved ARDS outcomes (208); however, no appropriate pharmacotherapy has emerged. A wide range of anti-inflammatory agents (e.g., corticosteroids, prostaglandins, n-acetylcysteine) had provided promising results in both rodent and large animal models, however without translational relevance, as clinical trials failed to achieve significant benefit for patients (209). In contrast, a recent study applied EVMP in human donor lungs, which were not acceptable for transplantation, to successfully establish a model of endotoxemia-derived lung injury via lipopolysaccharide (LPS) instillation into the pulmonary artery (210). Tightly reflecting the clinical setting, this approach resulted in a robust cytokine response, along with decreased pulmonary venous oxygen content over five hours as seen during ARDS. Supporting the concept of early pharmacological studies using EVMP, evaluating a novel small molecule, BC1215, which suppresses NF-κB signaling, in the ARDS model resulted in reduced induction of IL-1, IL-6, and IL-10 as measured by ELISA of BALF. RNA sequencing furthermore revealed that BC1215 administration after LPS exposure significantly blunted the NF-κB transcriptional response and preserved venous partial oxygen pressure (210).

In addition to evaluating the efficiency of pharmacotherapies, EVMP could furthermore support clinical phase 1 and 2 studies allowing for the delineation of organ-specific side effects, which are of crucial relevance for clinical transition. Commonly used animal models to test the hepatotoxicity of novel drugs, for example, display significant discrepancies from the human setting due to differences in drug metabolism and mostly homogeneous environmental and genetic conditions in inbred animal strains. In contrast, an EVMP model investigating the impact of acetaminophen-induced liver injury utilized human liver tissue from partial liver resections to mimic the clinical setting. Notably, the EVMP system allowed for hourly perfusate sampling and live assessment of clinical parameters showing compromised liver function during APAP poisoning with lower glucose consumption and lactate production rates while hepatocyte synthesis capacity had been preserved (211). Of note, evaluating liver function by clearance of indocyanine green revealed stable hepatocellular function during the entire perfusion period indicating a clinically relevant setting (211).

Finally, the utilization of EVMP models to initiate clinical trials could lower the ethical burdens of testing novel pharmacological drugs, thus accelerating the transition of promising candidates into the clinics (Figure 1H).

## 4. Outlook and limitations

EVMP—primarily applied in allogeneic organ transplantation—concomitantly provides an interface to investigate perfused organs in an almost physiological setting but with improved accessibility, which was the basic requirement to transfer and test out this technique in a broader spectrum. Applying *ex vivo* therapies to regenerate or cure diseased organs constitutes a feasible approach, which will be further expanded in the future for selected clinical indications thus having the potential to minimize the gap between demand and supply in organ transplantation. This can be implemented on the one hand by the improvement of otherwise discarded, marginal donor organs, in particular from donors after DCD and those retrieved from ECD (6). On the other hand, treating diseased organs *ex vivo* followed by autologous replantation of the cured organ may reduce the number of patients in need of organ transplantation (8–10). This autologous application should be limited to clinics that display profound experience in the field of temporary organ support systems and organ transplantation. It is noteworthy that, at the current state, the broad majority of studies investigating *ex vivo* supported organ reconditioning are still executed on a purely experimental level and derive from small study series.

Overall, there are two main factors to further advance this innovative research field in the future. At first, further improvements in preservation protocols enabling >24 h *ex vivo* perfusion or even more without significant organ damage need to be established. On the other hand, interdisciplinary exchange and cooperation need to look beyond the boundaries of the own professional discipline to learn and pick up ideas from related ones. The translation into the own clinical or research-associated area may enhance the welfare of patients, true to the saying “You dońt have to reinvent the wheel.”
